# Congenital rubella syndrome in Iran

**DOI:** 10.1186/1471-2334-5-44

**Published:** 2005-06-06

**Authors:** Jila Sadighi, Hasan Eftekhar, Kazem Mohammad

**Affiliations:** 1 Institute for Health Sciences Research, Tehran, Iran; 2 Tehran University of Medical Sciences, Tehran, Iran

## Abstract

**Background:**

Congenital rubella syndrome (CRS) can be prevented with appropriate vaccination programs. The prevalence rates of rubella and CRS in Iran are unknown; therefore, the risk of exposure in pregnant women is not clear. The prevalence of CRS in the pre-vaccine period can be estimated by evaluating the proportion of children in the population with sensorineural hearing loss attributable to rubella.

**Methods:**

This was a case-control study to estimate prevalence of CRS in Tehran (Iran) by evaluating the proportion of children with sensorineural hearing loss attributable to rubella. The study used rubella antibody titer as an indicator, and compared the prevalence of rubella antibody between children with and without sensorineural hearing loss. Using these findings, the proportion of cases of sensorineural hearing loss attributable to rubella was estimated.

**Results:**

A total of 225 children aged 1 to 4 years were entered into the study (113 cases and 112 controls). There was a significant difference between cases and controls with regard to rubella antibody seropositivity (19.5% vs. 8.9%, respectively, odds ratio = 2.47, 95% CI = 1.04–5.97). The proportion of sensorineural hearing loss cases attributable to rubella was found to be 12%, corresponding to a CRS prevalence of 0.2/1000.

**Conclusion:**

The prevalence of CRS was approximately 0.2/1000 before rubella vaccination in Iran, Moreover; the results suggest that implementation of appropriate rubella vaccination programs could potentially prevent about 12% of cases of sensorineural hearing loss in Iranian children. This data could potentially be used as baseline data, which in conjunction with an appropriate method, to establish a surveillance system for rubella vaccination in Iran. An appropriate surveillance system is needed, because the introduction of a rubella vaccine without epidemiological data and an adequate monitoring program could result in the shifting of rubella cases to higher ages, and increasing the incidence of CRS.

## Background

Rubella is a common, normally mild disease that mainly affects children aged 2–12 years. Rubella in pregnancy may cause abortion, stillbirth and congenital anomalies, or congenital rubella syndrome (CRS). Prior to the introduction of rubella vaccine in 1969, the disease was distributed evenly throughout the world. In temperate regions, the incidence was usually highest in late winter and early spring. Minor epidemics occurred every 6–9 years, with major epidemics occurring at intervals ranging from 10 to 30 years [1,2].

The rubella pandemic in the 1960's clearly demonstrated the extraordinary teratogenic potential of the rubella virus. In spite of the fact that 80% of pregnant women were immune to rubella in the United States, it is estimated that more than 12,500,000 cases of rubella occurred. Congenital rubella occurred in an estimated 30,000 pregnancies, with 10,000 resulting in fetal death or therapeutic abortion, and 20,000 resulting in infants born with CRS [3]. The estimated cost to the US economy was approximately $2 billion [4].

The incidence of congenital rubella varies in different populations and depends on the number of susceptible pregnant women, the circulation of rubella virus, and rubella vaccination coverage. According to the World Health Organization (WHO), at least 236,000 CRS cases occur in every non-epidemic year in developing countries, and this increase by up to 10 fold during epidemic years The CRS cases are rarely reported in these countries, and the extent of the problem remains unknown. However, the indiscriminate introduction of rubella vaccine without epidemiological data and an adequate monitoring program should be avoided because the occurrence of rubella cases can shift to higher ages and increase the incidence of CRS [5].

Rubella is often not notified, as many cases are not seen by a doctor or even recognized by the patient; consequently, rubella outbreaks can occur without clinical recognition. Nevertheless, studies in Central and South America, Africa, India and the Middle and Far East suggest that rubella is widespread and endemic in most developing countries [6,7].

The percentage of infection in the fetuses of mothers infected by rubella during the first trimester of pregnancy is greater than 80%. As a result, the target group for the vaccination is all women of childbearing age. Therefore, the fundamental reason for using the vaccine containing the rubella antigen is to prevent congenital rubella syndrome [8].

In October 2004, CDC convened an independent panel of internationally recognized authorities on public health, infectious disease, and immunization to assess progress toward elimination of rubella and congenital rubella syndrome in the United States, a national health objective for 2010. Since rubella vaccine licensure in 1969, substantial decline in rubella and CRS have occurred, and absence of endemic transmission in the United States is supported by recent data: fewer than 25 reported rubella cases each year since 2001, at least 95% vaccination coverage among school aged children, estimated 91% population immunity, adequate surveillance to detect rubella outbreaks and a pattern of virus genotypes consistent with virus originating in other parts of the world [9].

A rubella vaccination program in the United Kingdom (UK) was initiated in 1970. Reported cases of CRS declined from about 50 a year 1971–75 to just over 20 a year 1986–90, and rubella associated terminations from an average of 750 to 50 a year [10].

The European Region (EUR) of the World Health Organization (WHO) comprises 52 member countries, with an estimated population of 876 million. In 1998, the Regional Committee for EUR resolved to reduce the incidence of congenital rubella syndrome (CRS) in all countries to <1 per 100,000 live births by 2010. Large rubella outbreaks continue to occur in countries that only recently introduced rubella vaccination (e.g., Russian Federation and Romania). In many countries, CRS surveillance is not fully implemented, resulting in underestimates of CRS disease burden, both at country and regional levels [11]. The introduction of rubella vaccine in the infant immunization schedule should only be considered when coverage>80% can be assured on a long-term basis [12].

Between December 2003 and January 2004, the Ministry of Health and Medical Education of the Islamic Republic of Iran implemented a nationwide campaign to vaccinate about 32,000,000 people aged 5 to 25 years with a combined measles and rubella (MR) vaccine. Before this campaign, rubella vaccination was not included in the childhood vaccination schedule. After the campaign, the Ministry changed the childhood vaccination schedule so as to include 2 doses of the measles, mump and rubella (MMR) vaccine, one given at 15 months and the other at 4 to 6 years of age. Before the MR campaign in Iran, the epidemiology of rubella and congenital rubella was not clear. There is still no adequate surveillance system in place for congenital rubella after vaccination. An adequate surveillance system should monitor the following parameters: incidence of CRS, incidence of rubella, rubella immunity in women of childbearing age, rubella vaccination coverage and rubella outbreaks.

Retrospective studies on children with abnormalities similar to the complications of CRS in developing countries can estimate the rate of congenital rubella risk in these children. Cases of rubella-related deafness in children have been identified by comparing the prevalence of rubella antibody in children with and without sensorineural deafness. Therefore, a reduction in the number of deaf children has been used as an indication of reduced maternal rubella infection after the introduction of rubella vaccination programs [6]. Sensorineural hearing loss is one of the most common abnormalities (50%) associated with CRS [13]. Hearing loss present at birth is often not detected until a later age. Hearing loss can also occur as a delayed manifestation of CRS; and is the most common complication (80%) with late onset [14]. The hearing loss is bilateral, sensorineural type in all grades of severity [12].

Moreover, up to 50% of infections during pregnancy are sub-clinical, and many go unrecognized. Thus, the estimated incidence of rubella-related deafness (like the other CRS defects) is likely less than the true incidence [5]. Hearing impairment can result from fetal rubella not only during the first trimester, but also in the second and third trimesters of pregnancy [13]. Rubella serology is useful in epidemiological studies to examine the role of rubella as a cause of sensorineural hearing loss, because the number of acquired infections can be estimated from data gathered from controls and cases of rubella-related deafness in populations of children. The aim of this study was to use a similar method to estimate the prevalence of CRS by indicating the proportion of children in the population with sensorineural hearing loss attributable to rubella.

## Methods

A case-control study of 225 children aged 1–4 years was conducted from November 1995 to May 1996 in Tehran, Iran. The study compared the prevalence of rubella antibody between children with and without sensorineural hearing loss, and tested the hypothesis that congenital rubella is associated with an increased risk of sensorineural hearing loss.

The cases were 113 medically confirmed deaf children admitted to deaf educational centers. The controls were 112 children with normal hearing selected from the surgery ward of Amirkabir hospital in Tehran. One limitation of this study was the selection of the control group, because many parents with healthy children were reluctant to allow blood sampling of their children. Therefore, the control group was taken from among patients at a hospital surgery ward, excluding subjects who may have had an infectious disease.

It is significant that children under 1 and over 4 years of age were excluded from the study. In infants (mainly under 6 months), high levels of rubella antibody passively transferred from mother to fetus could result in an overestimate of seropositivity, while in children over 4 years old, rubella antibody is usually acquired from a postnatal infection. Detection of antibody in children 1 to 4 years of age was used to make a retrospective diagnosis of congenital rubella infection. It is also significant that rubella infection is most frequent among children 5 to 14 years of age [15].

During the time period covered by this study, rubella vaccination was not included in the national routine childhood vaccination; however, some physicians administered MMR vaccine instead of measles vaccine to their patients. Therefore, we excluded all children who had a history of MMR vaccination. History of MMR vaccination was obtained from the vaccination cards of children.

For all children, data were collected on maternal (prenatal and delivery) and neonatal histories. In addition, blood samples were taken from children and the titer of rubella antibody was determined. The serological technique employed was the hemagglutination inhibition (HI) test. Children with rubella HI antibody titers of 1:8 or greater were regarded as seropositive, and those with titers of less that 1:8 as seronegative. The Virology Department of the Public Health School (Tehran University of Medical Sciences) carried out the HI tests. Seropositive children in the case and control groups were compared using the chi-squared test and odds ratio (OR). The attributable risk (AR) was then estimated. The formula for calculating the AR using the odds ratio for disease in the exposed population is:

% Attributable Risk = *P (OR-1)/ 1+ P (OR-1)*

where P is the exposure prevalence in the controls as long as the disease is rare and the control group is reasonably representative of all non-cases in the population (proportion of rubella antibody in the control group), and OR is the odds ratio for disease in the exposed population.

Finally, we developed the following formula to estimate the prevalence of CRS:

"Prevalence of CRS = B * C * (1/D) * 1000"

This formula is derived from the following formula:

"Prevalence of CRS = [A * B * C * (1/D) * 1000] / A"

where A is the number of children during the year of study, B is the prevalence of hearing loss, C is the AR, and D is the frequency of sensorineural hearing loss following CRS.

## Results

There were 113 children in the case group and 112 children in the control group. The case and control groups consisted of 53% and 47% male subjects, respectively. This difference was not statistically significant (P = 0.2). No significant difference was found with regard to age distribution of cases as compared to controls (P = 0.12). The mean (SD) age was higher in cases than controls, but this difference was not significant: 2.9 years (SD = 1.1) compared with 2.6 years (SD = 1.3) (P = 0.4).

The percentage of children with rubella antibody in the case and control groups is shown in Table 1. The percentage of children with rubella antibody was significantly higher in cases than controls: 19.5% compared with 8.9%, respectively (OR = 2.47, 95% CI 1.04–5.97, P = 0.02). Therefore, congenital rubella was associated with a significantly increased (greater than two times) sensorineural hearing loss in children. The AR was estimated to be about 12%, which indicates that about 12% of the children born with hearing loss were damaged as a result of congenital rubella, and thus that it may be possible to reduce the incidence of sensorineural hearing loss by up to 12% by preventing rubella infection in pregnant women.

**Table 1:** Rubella antibody status in hearing impaired (case) and normal control children aged 1–4 years, Tehran, Iran

**Rubella antibody status**	**Case n (%)**	**Control n (%)**	**Odds Ratio (95% CI)**
Seropositive	22 (19.5)	10 (8.9)	
Seronegative	91 (80.5)	102 (91.1)	2.47 (1.04–5.94)
Total	113 (100)	112 (100)	

According to the results of this study, the prevalence of CRS in Tehran was estimated to be about 0.2/1000 children. This prevalence was estimated using the following data:

A = number of children aged 1 – 4 years in Tehran (1995): 775000 (using national data)

B = prevalence of hearing loss in children: 1/1000 (using national data)

A*B = children with hearing loss: 775 (expected)

C = attributable risk (derived from the present study): 12%

A*B*C = children with rubella-related deafness: 93 children (expected)

D = frequency of sensorineural hearing loss associated with CRS: 50% (derived from previous studies)

A*B*C*1/D = the number of children with CRS: 186

Therefore:

Prevalence of CRS in Tehran = 186 / 775000 = 0.2/1000

Considering the number of children aged 1 – 4 years in Iran and the prevalence of CRS estimated here (0.2/1000 children), the present results suggest that tens of CRS cases could be prevented each year by appropriate vaccination programs.

Maternal history in sensorineural hearing-impaired children (case group) according to rubella antibody status is shown in Table 2. Nine mothers (41%) of 22 deaf seropositive children reported a history of rubella infection, rash, or rubella exposure during pregnancy, whereas none of 91 mothers of seronegative deaf children reported these events (p = 0.000).

**Table 2:** Rubella antibody status in sensori-neural hearing-impaired children aged 1–4 years according to maternal history of rubella infection, Tehran, Iran

**Maternal history of rubella**	**Seropositive n (%)**	**Seronegative n (%)**	**P-value**
Positive	9 (41)	0 (0)	
Negative	13 (59)	91 (100)	0.000
Total	22 (100)	91 (100)	

## Discussion

The present findings indicate that the prevalence of CRS in Iran is approximately 0.2/1000 (Before rubella vaccination in Iran). World Health Organization reported that in the absence of widespread rubella vaccination, the incidence of CRS varies between 0.1–0.2 per 1,000 live births, with higher rates (1–4 per 1,000 live births) during epidemics [2].

During rubella outbreaks, rates of CRS per 1000 live births were at least 1.7 in Jamaica, 0.7 in Oman, 2.2 in Panama, 1.5 in Singapore, 0.9 in Sri Lanka, and 0.6 in Trinidad and Tobago. These rates are similar to those reported from industrialized countries during the pre-vaccine era [16].

The prevalence of bilateral sensorineural hearing loss is 0.5 to 1 newborns per 1000 live births. In addition, the onset of hearing loss can occur at any time throughout childhood. Thus, it is estimated that the prevalence of bilateral hearing loss increases to 1.5–2/1000 children under the age of 6 years [15]. In Iran, the prevalence of hearing loss is 1/1000 children [17]. Considering the prevalence of sensorineural hearing loss, the estimated AR in this study (12%), it is estimated that congenital rubella was the cause of deafness in approximately 93 children aged 1–4 in Tehran in the year considered in the present study. If we assume that the epidemiology of rubella in Tehran, the capital city of Iran, is similar to that in other areas of Iran, we obtain the estimate that in the year of the present study there were approximately 620 children (1–4 years) in Iran with deafness that could have been prevented by rubella vaccination.

Some investigations in Iran showed that rubella immunity in women of childbearing age from 1968 through 1995 (the time of this study) fluctuated between 70% and 95% [18-22]. The rate of rubella immunity in this population in 1995 was estimated as 80% [23]. Therefore, the year considered in the present study was a non-epidemic year, and the number of children born with deafness due to rubella would be expected to increase in epidemic years.

Nine mothers (41%) of 22 deaf, seropositive children in the present study reported a history of rubella, rash, or rubella exposure during pregnancy. Other studies have also reported between 40% and 75% of deaf seropositive children had such a maternal history [24].

In this study, the degree of hearing loss in children who attended deaf educational centers was often higher than 50 dB (severe to profound hearing loss), and their hearing loss was bilateral. Thus, children with low severity of deafness were not included in this study, and the relation between severity of deafness and congenital rubella could not be estimated.

About 20% of the children in deaf educational centers were not included because of a history of MMR (Measles, Mump and Rubella) vaccination, which may lead to an underestimation of the OR. Meanwhile, experience in other countries suggests that if MMR vaccination coverage is less than 60%–70%, it may actually increase the age of infection, and therefore the incidence of CRS [1].

After comparing vaccination cards with parents' reports about vaccination history, it was found that five deaf children among the cases actually had a history of MMR vaccination, and they were consequently excluded from the study. Contrary to expectations, rubella antibody titer was negative in two of these children. Therefore, the efficacy of MMR vaccine should be investigated in future studies.

According to research done in Iran (during the year of this study), the rate of rubella immunity has reached about 80% [23]; however, this rate of immunity is similar to that in other countries during the rubella pandemic of the 1960's, which claimed thousands of victims [3,4]. Epidemiological evidence has shown that while rubella virus continues to circulate among children, there is still a risk of infection in pregnant women, even though only 3% of them are non-immune, and there is little prospect of eliminating CRS [1,25].

The world has now cumulated 35 years of lessons on use of rubella vaccine, and some striking examples of how rubella vaccination strategies should and should not be applied [10,11,26-31]. Most importantly, studies in developed countries have generated the following recommended vaccination program: routine MMR vaccination at 12–15 months of age followed by a second dose of MMR vaccine at 4–6 years (both sexes) [10,32,33]. This study clearly showed the necessity for suitable rubella vaccination program in Iran. However, inadequately implemented childhood vaccination runs the risk of altering rubella transmission dynamics and can lead to increase insusceptibility in women of childbearing age with the potential of increased numbers of cases of CRS. Consequently, it is essential that childhood vaccination programs achieve and maintain high levels of coverage [12].

Before the MR campaign in Iran, the epidemiology of rubella and congenital rubella was not clear. In spite of this unclear epidemiology, rubella vaccination was launched in Iran (after the time period covered by the present study). Now, due to limited disease surveillance and reporting systems, data on the incidence of rubella and CRS in Iran are scant. This study provides data on the prevalence of CRS in Iran; this data could potentially be used as baseline data, which in conjunction with an appropriate method, to establish a surveillance system for rubella vaccination in Iran.

## Conclusion

Rubella is a common communicable viral disease of childhood, and rubella in pregnancy may cause CRS. Sensorineural hearing loss is the most common abnormality associated with CRS, and it is the most common complication with late onset. The present findings indicate that the prevalence of CRS was approximately 0.2/1000 before rubella vaccination in Iran, Moreover; the results suggest that implementation of appropriate rubella vaccination programs could potentially prevent about 12% of cases of sensorineural hearing loss in Iranian children. This data could potentially be used as baseline data, which in conjunction with an appropriate method, to establish a surveillance system for rubella vaccination in Iran. An appropriate surveillance system is needed because the introduction of rubella vaccine without epidemiological data and an adequate monitoring program may result in the shifting of rubella cases to higher ages, and increased incidence of CRS.

## List of abbreviations

**CRS: **Congenital Rubella Syndrome

**AR: **Attributable Risk

**OR: **Odds Ratio

**HI: **Heamagglutination Inhibition

**SD: **Standard Deviation

## Competing interests

The author(s) declare that they have no competing interests.

## Authors' contributions

**JS: **Study idea, literature review, methodology development, data analysis and interpretation, and writing the final article.

**HE: **Supervisor of the research project.

**KM: **Counseling the methodology and supervised the statistical analysis.

## Pre-publication history

The pre-publication history for this paper can be accessed here:



## References

[B1] Galazka A (1991). Rubella in Europe. Epidemiol Infect.

[B2] World Health Organization (2000). Rubella vaccines. WHO Position Paper. Wkly Epidemiol Rep.

[B3] Herrmann KL (1991). Rubella in the United States: toward a strategy for disease control and elimination. Epidemiol Infect.

[B4] Preblud SR, Serdula MK, Frank JA, Brandling-Bennett AD, Hinman AR (1980). Rubella vaccination in the United States: a ten years review. Epidemiol Rev.

[B5] World Health Organization (EPIGAG) (1991). Rubella and congenital rubella syndrome in developing countries 14th Meeting.

[B6] Miller CI (1991). Rubella in the developing world. Epidemiol Infect.

[B7] Mingle JAA (1985). Frequency of rubella antibodies in the population of some African countries. Rev Infect Dis.

[B8] Pan American Health organization. Introduction of new vaccines in the national vaccination. MMR vaccine. http://www.paho.org/english/sha/epibul_95-98/be961vac.htm.

[B9] Centers for Disease Control and Prevention (CDC) Achievements in public health: Elimination of rubella and congenital rubella syndrome – United States, 1969–2004. MMWR.

[B10] Tookey P Rubella in England, Scotland and Wales. Euro Surveill.

[B11] Centers for Disease Control and Prevention (CDC) Progress toward elimination of measles and prevention of congenital rubella infection – European region, 1990–2004. MMWR.

[B12] Report of a meeting on preventing congenital rubella syndrome: immunization strategies, surveillance needs. Geneva, 12–14 January 2000. http://www.who.int/vaccines-documents/DocsPDF00/www508.pdf.

[B13] Northern JL, Downs MP (1991). Hearing in Children.

[B14] Nelson WE, Behrman RE, Kliegman RM, Arvin AM (1992). Nelson Textbook of Pediatrics.

[B15] Nelson WE, Behrman RE, Kliegman RM, Arvin AM (1996). Nelson Textbook of Pediatrics.

[B16] Cutts FT, Robertson SE, Diaz-Ortega JL, Samuel R (1997). Control of rubella and congenital rubella syndrome (CRS) in developing countries, Part 1: Burden of disease from CRS. Bull World Health Organ.

[B17] Ministry of Health and Medical Education (1990). Health and Disease in Iran. National report.

[B18] Naficy K, Saidi S (1970). Serological survey on viral antibodies in Iran. Tropical and geographical medicine.

[B19] Saidi S (1972). Epidemiological survey of rubella immunity in Iran. Bulletin of the World Health Organization.

[B20] Kabiri M, Moattari A (1993). The rubella immunosurveillance of Iranian females: an indication of the emergence of rubella outbreak in Shiraz, Iran. Iranian journal of medical sciences.

[B21] Modarres S, Modarres S, Oskoii NN (1996). The immunity of children and adult females to rubella virus infection in Tehran. Iranian journal of medical sciences.

[B22] Pakzad P, Ghafourian M (1996). Rubella survey among pregnant women and congenitally infected infants in Khouzestan province. Medical journal of Ahwaz.

[B23] Rahimi F, Sarafnegad A, Salarbehzadi SH (1994). Survey of rubella immunity in Tehran. Thesis.

[B24] Gumpel SM, Hayes K, Dudgeon JA Congenital perceptive deafness: role of intrauterine rubella. Br Med J.

[B25] Miller E (1991). Rubella in the United Kingdom. Epidemiol-Infect.

[B26] Levy-Bruhl D, Six C, Parent I Rubella control in France. Euro Surveill.

[B27] Katow S Surveillance of congenital rubella syndrome in Japan, 1978–2002: effect of revision of the immunization law. Vaccine.

[B28] Davidkin I, Peltola H, Leinikki P Epidemiology of rubella in Finland. Euro Surveill.

[B29] Ciofi Degli Atti M, Filia A, Revello M, Buffolano W, Salmaso S Rubella control in Italy. Euro Surveill.

[B30] Rafila A, Marin M, Pistol A, Nicolaiciuc D, Lupulescu E, Uzicanin A, Reef S A large rubella outbreak, Romania – 2003. Euro Surveill.

[B31] Panagiotopoulos T, Georgakopoulou T Epidemiology of rubella and congenital rubella syndrome in Greece, 1994–2003. Euro Surveill.

[B32] American Academy of Pediatrics Committee on Infectious Diseases (2005). Recommended childhood and adolescent immunization schedule: United States, 2005. Pediatrics.

[B33] Centers for Disease Control and Prevention (CDC) Recommended childhood and adolescent immunization schedule -United States, 2005. MMWR.

